# Nevirapine inhibits migration and invasion in dedifferentiated thyroid cancer cells

**DOI:** 10.1111/1759-7714.13211

**Published:** 2019-10-21

**Authors:** Hongxia Shang, Junyu Zhao, Jinming Yao, Huanjun Wang, Shengnan Wang, Jianjun Dong, Lin Liao

**Affiliations:** ^1^ Division of Endocrinology, Department of Internal Medicine Shandong Provincial Qianfoshan Hospital, Shandong University Jinan China; ^2^ Division of Endocrinology, Department of Internal Medicine Shandong Provincial Qianfoshan Hospital, the First Hospital Affiliated with Shandong First Medical University Jinan China; ^3^ Division of Endocrinology, Department of Internal Medicine Qilu Hospital of Shandong University Jinan China

**Keywords:** Dedifferentiated thyroid cancer, IL‐6/STAT3 signaling pathway, invasion, migration, nevirapine

## Abstract

**Background:**

Metastatic or recurrent thyroid cancer often behaves aggressively, and approximately two‐thirds of patients present with radioiodine resistance. Effective therapies to suppress thyroid cancer metastasis are urgently needed. Nevirapine has been proved to suppress tumor growth and induce differentiation in several tumor cells, but has not previously been evaluated in metastasis of thyroid cancer. The present study aimed to investigate the effect of nevirapine on migration and invasion in dedifferentiated thyroid cancer cells.

**Methods:**

Human dedifferentiated thyroid cancer cell line (WRO 82‐1) was subject to real‐time quantitative PCR, western blot and transwell migration/invasion assays. The liver metastasis in tumor xenografts of nude mice was subject to hematoxylin‐eosin (HE) staining.

**Results:**

Nevirapine significantly repressed cell migration and invasion in WRO 82‐1 cells, and surprisingly significantly decreased liver metastatic tumor in the nude mouse model of dedifferentiated thyroid cancer compared with that of the control. Moreover, nevirapine significantly decreased the expression of IL‐6 mRNA and phosphorylation of JAK2 (Y1007+Y1008) and STAT3 (Tyr 705) in WRO 82‐1 cells compared with those in control cells.

**Conclusion:**

Our findings suggest that nevirapine significantly repressed migration and invasion/metastasis in WRO 82‐1 cells and tumor xenografts, which may be related to inhibition of IL‐6/STAT3 signaling pathway. It promises great potential as a novel therapy for thyroid cancer, especially for those patients with metastasis.

## Introduction

Thyroid cancer is one of the fastest growing cancers worldwide.^1^, ^2^ It has two outstanding features. The first is the high rate of metastasis to lymph nodes or other organs, which is as high as 30% to 59.1%.^1^, ^3^, ^4^, ^5^ The second is the resistance to chemotherapy and conventional external beam radiotherapy.^6^, ^7^ Metastatic thyroid cancer behaves aggressively and about two‐thirds of patients with metastatic disease ultimately present with radioiodine refractory disease.^8^, ^9^ Once it metastasizes to other organs it is hard to treat and therefore it is important to exploit new therapeutic agents which are effective in the treatment of metastatic thyroid cancer.

Nevirapine, the first non‐nucleoside reverse transcriptase inhibitor approved by the US Food and Drug Administration (FDA) in 1996, is an important component of highly active antiretroviral therapy widely prescribed for human immunodeficiency virus‐1 (HIV‐1) infection.^10^, ^11^, ^12^ Recently, accumulating findings have shown that nevirapine acts as a cytostatic and differentiating agent by modulating gene expression in progenitor cells and several human cancers, such as melanoma, prostate cancer, lung cancer, colon cancer, acute myeloid leukemia (AML) and so on.^13^, ^14^, ^15^, ^16^, ^17^ In our previous study, nevirapine promoted the mRNA expression of sodium iodide symporter (NIS) in anaplastic thyroid cancer. However, there has not previously been any study published on the effects of nevirapine on cell migration and invasion/metastasis in thyroid cancer.

Tumor migration and invasion/metastasis is a complicated process and is often correlated with extracellular matrix hydrolysis, a process mediated by several proteolytic enzymes, including MMPs which are proteinases involved in the migration and invasion of malignant cells.^18^ There are more than 20 MMPs involved, among these MMPs, MMP2 and MMP9 are pivotal for the invasion and metastasis of cancers.^19^, ^20^, ^21^

The role of the Janus kinase/signal transducer and activator of transcription 3 (JAK/STAT3) pathway is a well‐known mediator of tumorigenesis in different tumors, and is associated with migration and invasion of cancers by increasing expression of MMP2 and MMP9.^22^, ^23^, ^24^ Phosphorylation of STAT3 on Tyr705 is the major mechanism of STAT3 activation, which is mediated by JAK upon stimulation of the heterodimeric gp130/cytokine‐specific receptor complex by the IL‐6 family of cytokines, including IL‐6.^25^

The present study was undertaken to explore the effects of nevirapine on cell migration and invasion/metastasis and the underlying mechanism in WRO 82‐1 cells, which was derived from a metastatic lesion of a patient with follicular thyroid carcinoma^26^, ^27^, ^28^ and in athymic mouse xenografts.

## Methods

### Cell culture

The human dedifferentiated thyroid carcinoma cell WRO 82‐1 was purchased from Sigma‐Aldrich (Munich, Germany) and cultured in RPMI 1640 medium (Gibco, USA) supplemented with 10% fetal bovine serum (FBS; Gibco, USA) at 37°C in a humidified atmosphere with 5% CO_2_. Nevirapine (purity, ≥99%) was purchased from the National Institute of Control of Pharmaceutical and Biological Products in China, dissolved in dimethyl sulfoxide (DMSO) to prepare a 250 mM stock solution and then diluted in medium immediately before use. The cells were treated with various concentrations of nevirapine (100 and 200 μM) prior to being subjected to various assays. In this study, negative control cells (0 μM) were treated with culture medium containing 0.1% DMSO. Incubation was carried out continuously for 72 hours.

### Transwell migration assay

WRO 82‐1 cells were treated with 200 μM nevirapine for 72 hours. The migration assay was carried out according to the manufacturer's protocol. Briefly, migration chambers (Corning, USA) were placed in a 24‐well plate, then 200 μL of WRO 82‐1 cells (2.5 × 10^5^/mL in serum‐free medium) were seeded on each transwell filter with 8 μM porosity and 750 μL culture media with 10% FBS added in the lower chamber. The cells were then incubated at 37°C in a humidified atmosphere with 5% CO_2_ for 24 hours, washed twice in PBS, fixed with 4% formaldehyde at room temperature for 15 minutes, permeabilized by 100% methanol at room temperature for 20 minutes, and then stained with crystal violet for 20 minutes at room temperature. The nonmigrated cells were scraped off with cotton swabs. Cells that had migrated through the pores were counted in five randomly selected fields under a light microscope at 20× objective magnification. The experiments were performed in triplicate.

### Transwell invasion assay

Cell invasion was assessed using transwell cell culture chambers, according to the manufacturer's protocol. We used 24‐well cell culture inserts (Corning, USA) with a polyethylene terephthalate membrane (8 μM pores). The membranes of each upper chamber were coated with Matrigel (100 μg/cm^2^; BD) and then incubated at 37°C overnight for gelling. Before each assay, the WRO 82‐1 cells were treated with nevirapine at the indicated concentration for 72 hours. For cell invasion assay, 200 μL of WRO 82‐1 cells (5 × 10^5^/mL in serum‐free medium) were seeded in the upper chamber in serum‐free medium, and 750 μL of media supplemented with 10% FBS was added to the lower chamber. The cells were then incubated at 37°C for 48 hours, washed twice with PBS, fixed with 3.7% formaldehyde at room temperature for two minutes, permeabilized by 100% methanol at room temperature for 20 minutes, and then stained with crystal violet for 15 minutes at room temperature. The cells that adhered to the upper surface of the chamber were carefully removed using cotton swabs, and those on the bottom surface of the membrane were imaged, after which cells in five randomly selected fields were counted under a light microscope (Leica, Germany) at 20× objective magnification. Experiments were performed in triplicate.

### Real‐time quantitative PCR

Total RNA was extracted using TriZol reagent (TaKaRa, Japan), according to the manufacturer's instructions, and then subjected to reverse transcription to synthesize cDNA using the First Strand cDNA Synthesis Kit (TaKaRa, Japan). Quantitative real‐time PCR was performed with SYBR Green (GenStar, China) with an ABI PRISM 7500 Real‐time PCR System (Applied Biosystems, USA). Target gene expression levels were calculated using ΔΔCt and comparative methods after being normalized to GAPDH expression levels. The primer sequences were as follows: IL‐6 forward 5′‐ATG TAG CCG CCC CAC ACA GA‐3′, reverse 5′‐GCA TCC ATC TTT TTC AGC CAT C‐3′; VEGFA forward 5′‐AGG GCA GAA TCA TCA CGA AGT‐3′, reverse 5′‐AGG GTC TCG ATT GGA TGG CA‐3′; MMP‐2 forward 5′‐ CTG GGA GCA TGG CGA TGG ATA‐3′, reverse 5′‐ GGA AGC GGA ATG GAA ACT TG‐3′; MMP‐9 forward 5′‐ GCC ATG TCT GCT GTT TTC TAG AGG‐3′, reverse 5′‐ CAC ACT CCA GGC TCT GTC CTC TTT‐3′; and GAPDH forward 5′‐CAG AAC ATC ATC CCT GCC TCT AC‐3′, reverse 5′‐ TTG AAG TCA GAG GAG ACC ACC TG‐3′.

### Western blot analysis

The cells were washed with PBS after being treated with nevirapine for 72 hours, and then lysed with RIPA buffer containing protease inhibitors cocktail 1× protease inhibitor cocktail (100:1). The lysate was centrifuged at 12 000 rpm for 20 minutes to remove debris. The supernatant was diluted with 5× loading buffer (4:1) and denatured in a boiling water bath (95°C) for 5 minutes, then preserved at −80°C. Protein concentrations were estimated by BCA assay. The 30 μg of protein extract was separated by SDS‐PAGE and then transferred to PVDF membranes, which were treated with 5% nonfat dry milk for one hour at room temperature. The membranes were later probed with the following primary antibodies overnight at 4°C, according to the manufacturer's protocol: rabbit polyclonal anti‐MMP2 (1:500, Affinity #AF0577, USA), rabbit monoclonal anti‐MMP9 (1:1000, Abcam #ab38898, UK), rabbit monoclonal anti‐JAK2 (1:2000, Abcam #ab108596), rabbit monoclonal anti‐pJAK2 (Y1007+Y1008) (1:1000, Abcam #ab32101), rabbit monoclonal anti‐pSTAT3 (Tyr705) (1:1000, Abcam #ab76315,UK), rabbit monoclonal anti‐STAT3 (1:2000, Abcam #ab68153, UK), VEGF (1:500, Affinity #AF5131, USA) and rabbit polyclonal anti‐GAPDH (1:5000, Proteintech #10494‐1‐AP, China). The membranes were then washed and incubated with HRP‐conjugated secondary anti‐rabbit IgG antibody (1:10000, Proteintech #SA00001‐2, China) for one hour at room temperature, after which the blots were detected by an enhanced ECL chemiluminescence system (Millipore, USA). The quantification of band intensity was performed using ImageJ software (Rawak Software, Germany).

### Tumor xenografts and animal treatment

A total of 10 four‐week‐old BALB/c (nu/nu) male nude mice (Vital River, China) were randomly assigned to two groups, kept in accordance with Chinese community guidelines. Mice were inoculated subcutaneously with WRO 82‐1 cells (1 × 10^6^/mouse) suspended in 300 μL PBS. Mice in the treated group were treated orally five days a week with nevirapine (150 mg/kg/day) in DMSO freshly diluted 1:4 with physiological solution, and the negative control mice were treated with vehicle (physiological solution with 20% DMSO). Treatment was started 10 days after tumor implant (tumor xenograft larger than 5 mm). Three weeks after tumor implant, animals were sacrificed and their tumors were dissected. All applicable international, national, and/or institutional guidelines for the care and use of animals were followed and approved by the Ethics Committee of Qianfoshan Hospital, Shandong University.

### Hematoxylin‐eosin (HE) staining

The tissues were immersion‐fixed in 4% paraformaldehyde for 24 hours, embedded in paraffin, and cut into 4 μM slices. After deparaffinization and rehydration, the sections were stained with HE staining to observe the morphology by microscope (Leica, Heidelberg, Germany).

### Statistical analysis

All data were expressed as mean ± SD and analyzed with GraphPad Prism 5 (GraphPad Software, California, USA). Student's *t*‐test was used to evaluate the statistical difference between groups in SPSS 20.0 software (SPSS, Inc., Chicago, USA). A *P*‐value of <0.05 was considered statistically significant.

## Results

### Nevirapine significantly reduced metastasis in the liver of athymic mice

**Figure 1:**
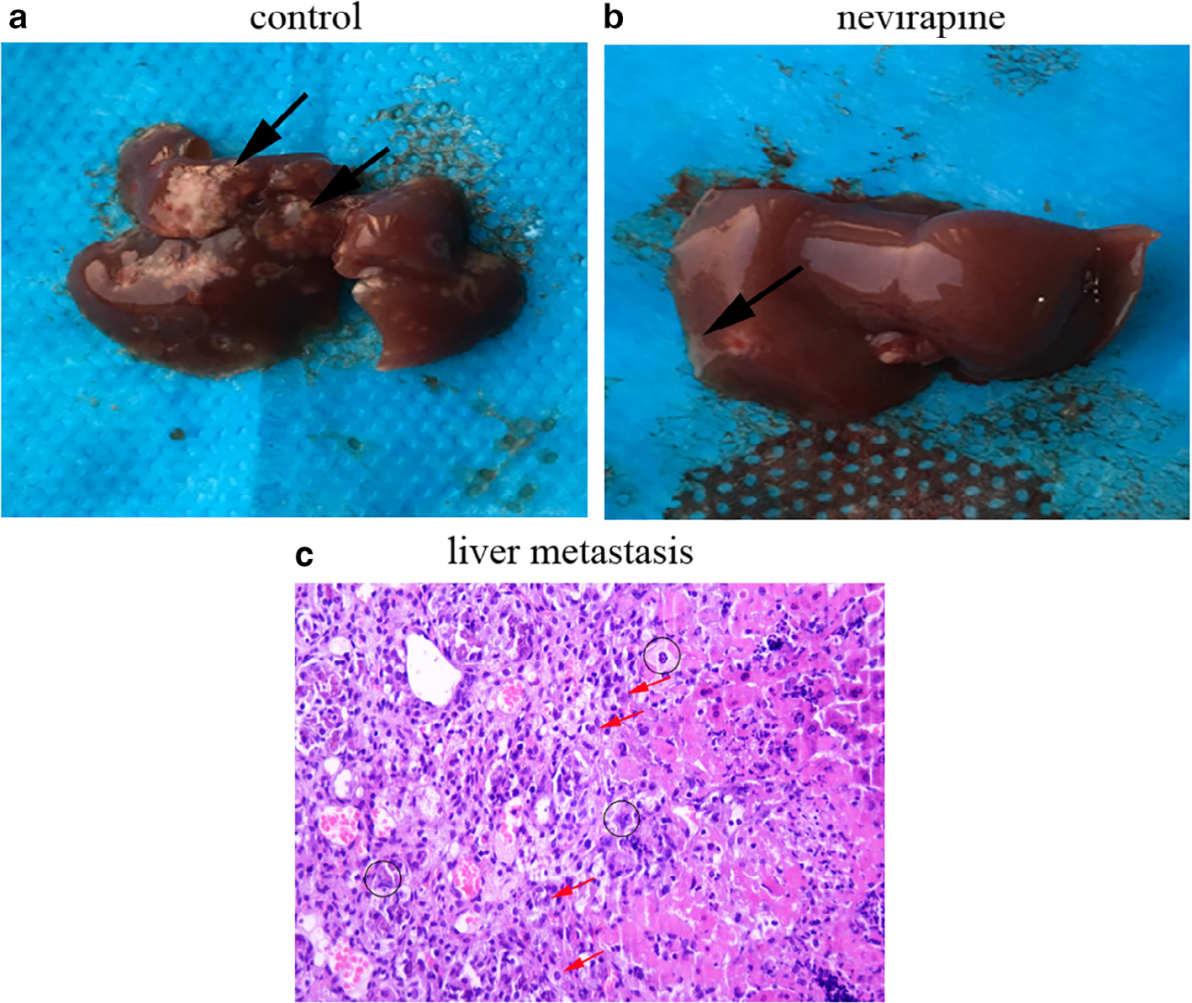
Nevirapine suppresses thyroid cancer cell metastasis in vivo. (**a**) The livers of control mice and (**b**) nevirapine‐treated mice were inspected for metastases. (**c**) The cell morphology was observed by HE staining. Thyroid cancer metastases are indicated by arrows and circles.

After inoculation of WRO 82‐1 cells in BALB/c nude mice, the animals received nevirapine treatment. Three weeks after treatment, we were surprised to see that the tumor metastasized to liver (Fig 1b) was reduced significantly compared with that of the control (Fig 1a), and the larger metastases were accompanied by necrosis in the liver of the control group. To further confirm the above observation, TTF‐1 and TG, two thyroid‐specific indicators were detected by immunohistochemistry, but the results were negative which may be related to the less expression of both proteins in dedifferentiated thyroid cancer. Subsequently, in order to further confirm whether metastases were derived from dedifferentiated thyroid cancer, HE staining, to observe cell morphology, was performed. The result in Figure 1c presented the spindle cells accompanied by a large number of pathological mitotic figures (arrow) and atypical giant cells (circle), which are the pathological features of dedifferentiated thyroid cancer. From the above we can see that dedifferentiated thyroid cancer metastasized to the liver and nevirapine inhibited the metastasis. In addition, it should be noted that although thyroid cancer is most likely to metastasize to the lungs, we did not find metastases by visual inspection and HE staining.

To further explore the mechanism, we conducted an in vitro study with the dedifferentiated thyroid cancer cell line WRO 82‐1.

### Nevirapine inhibited the migration of WRO 82‐1 cells

**Figure 2:**
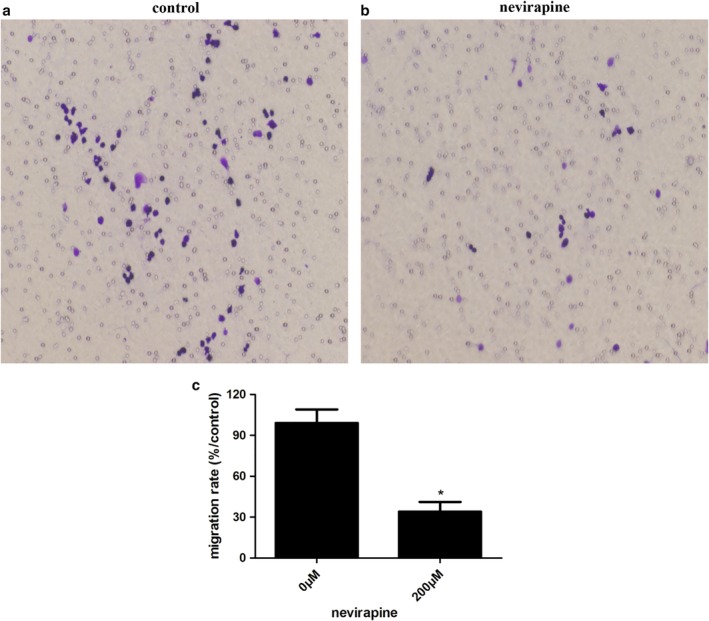
Nevirapine inhibited WRO 82‐1 cell migration. (**a**) Cell migration was detected by transwell migration assay. (**b**) The percentage of migration cells is shown as the mean ± SD of five replicate experiments. *P < 0.05.

To evaluate the effect of nevirapine on the migration of dedifferentiated thyroid cancer cells, we performed transwell migration assay. WRO 82‐1 cells were cultured and then incubated with different doses (0, 100 and 200 μM) of nevirapine for 72 hours. Treatment with various doses of nevirapine for 72 hours significantly decreased the migration rates of cells. As shown in Figure 2, migration rates of WRO 82‐1 cells receiving 200 μM nevirapine were significantly decreased to 34.38 ± 2.35% compared with the cells without nevirapine with statistical significance (*P* < 0.001). These data showed that nevirapine inhibited cell migration in the dedifferentiated cell line.

### Nevirapine inhibited the invasion of WRO 82‐1 cells

**Figure 3:**
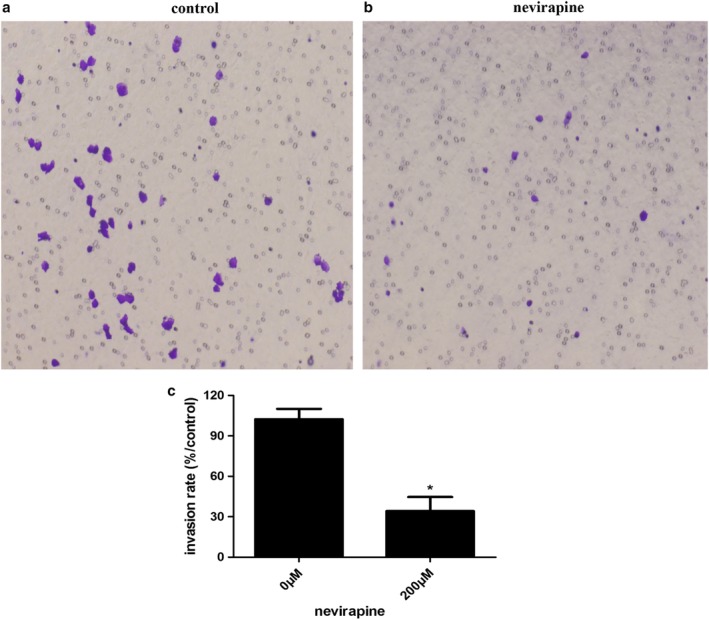
Nevirapine inhibited WRO 82‐1 cell invasion. (**a**) Cell invasion was detected by transwell invasion assay. (**b**) The percentage of invasion cells is shown as the mean ± SD of five replicate experiments. *P < 0.05.

The capacity of cell invasion was performed with a Matrigel‐coated filter. As shown in Figure 3, the invasion rates were significantly decreased to 31.47 ± 2.76% in WRO 82‐1 cell treated with 200 μM nevirapine for 72 hours compared with those of untreated cells, respectively. The results indicated that the ability of traversing Matrigel‐coated layer was decreased significantly (*P* < 0.001) in nevirapine‐treated WRO 82‐1 cells compared with that of untreated cells, suggesting that nevirapine significantly inhibited the invasion of WRO 82‐1 cells.

### Nevirapine downregulated expression of both mRNAs and proteins related to migration and invasion in WRO 82‐1 cells

**Figure 4:**
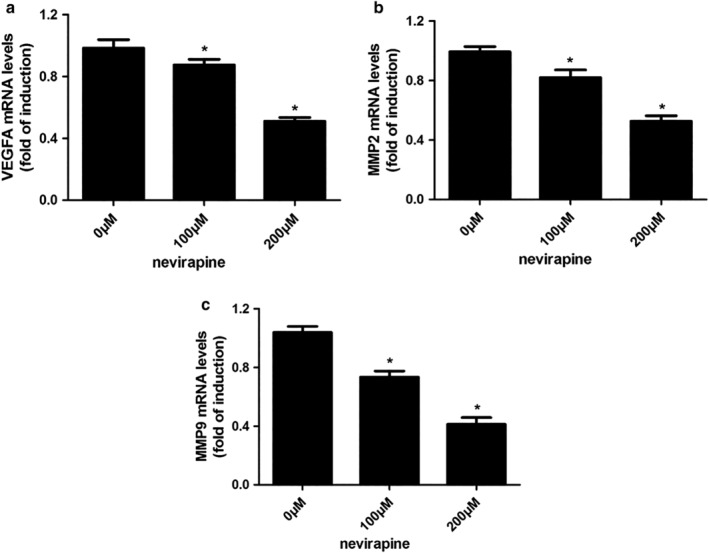
Nevirapine downregulated the expression of mRNAs related to migration and invasion in WRO 82‐1 cells. Changes of mRNA expression of (**a**) VEGFA, (**b**) MMP2 and (**c**) MMP9 were detected by quantitative real‐time PCR. *n* = 3, *P < 0.05.

**Figure 5:**
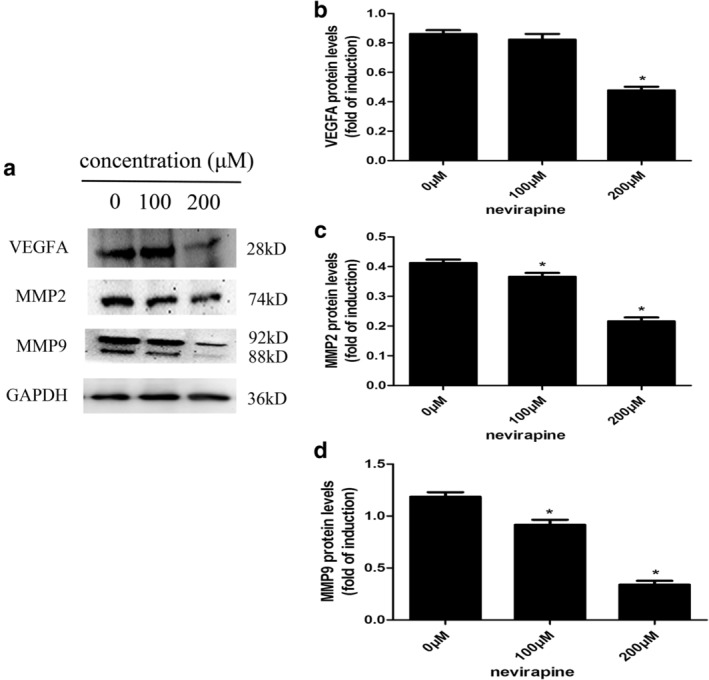
Nevirapine downregulated the expression of proteins related to migration and invasion in WRO 82‐1 cells. (**a**) Changes of protein expression of protein expression of VEGFA, MMP2 and MMP9 were determined by western blotting. The histograms show the results of the quantitative analysis of changes in (**b**) VEGFA, (**c**) MMP2 and (**d**) MMP9 protein expression. *n* = 3, *P < 0.05.

Several proteins are closely related to the migration and invasion of cancers, such as VEGFA, MMP2, and MMP9. Therefore, we assessed the effects of nevirapine on the mRNA and protein expression of these proteins by quantitative real‐time PCR (Fig 4) and western blotting (Fig 5), respectively. WRO 82‐1 cells were treated with nevirapine of 0, 100 and 200 μM for 72 hours. The results demonstrated that treatment with 100 and 200 μM nevirapine significantly decreased VEGFA, MMP2 and MMP9 expression in a dose‐dependent manner in WRO 82‐1 cells compared with untreated controls. WRO 82‐1 cells treated with nevirapine (Fig 4) had a significantly lower mRNA expression of VEGFA, MMP2 and MMP9 compared with cells treated with 0 μM nevirapine (all *P* < 0.05). Nevirapine also significantly decreased the protein expression of VEGFA, MMP2 and MMP9 compared with cells treated with 0 μM nevirapine (except the protein expression of VEGFA in 100 μM nevirapine‐treated cells, all *P* < 0.05; Fig 5).

### Possible mechanisms by which nevirapine inhibited the migration and invasion of thyroid cancer by inhibiting IL‐6/STAT3 signaling pathway

**Figure 6:**
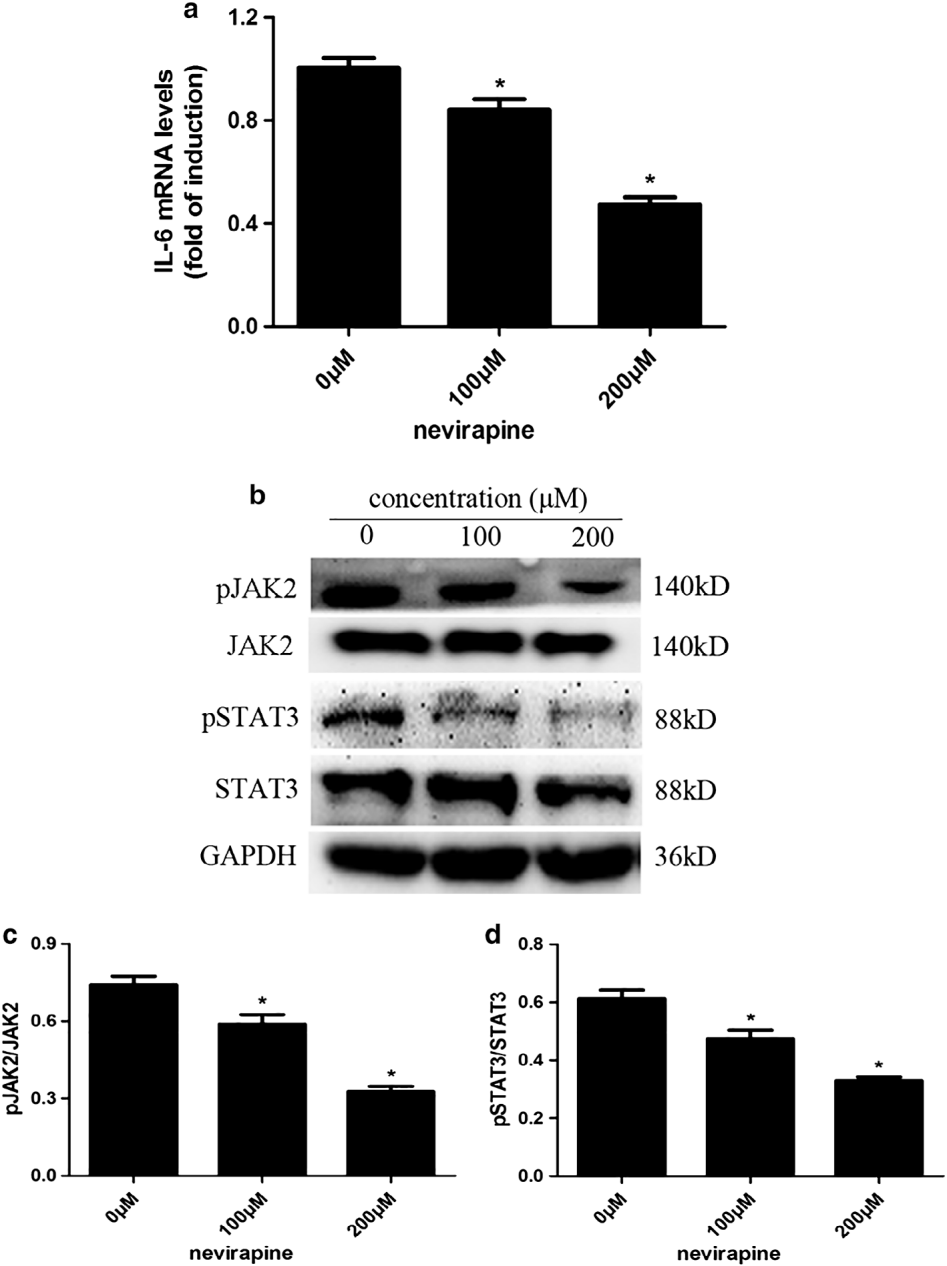
Nevirapine inhibited IL‐6/STAT3 signaling pathway. (**a**) The expression of IL‐6 mRNA was tested by quantitative real‐time PCR. (**b**) The phosphorylation of JAK2 (Y1007+Y1008) and STAT3 (Tyr705) was determined by western blot analysis and represented by the ratios of (**c**) pJAK2 (Y1007+Y1008)/JAK2 and pSTAT3 (Tyr705)/STAT3. *n* = 3, *P < 0.05.

The effects of nevirapine on mRNA expression of IL‐6 were assessed by quantitative real‐time PCR (Fig 6a) and protein expression of pJAK2 (Y1007+Y1008)/JAK2 and pSTAT3 (Tyr705)/STAT3 by western blot (Fig 6b). The results showed that treatment with 100 and 200 μM nevirapine for 72 hours significantly decreased mRNA expression of IL‐6 and phosphorylation of JAK2 (Y1007+Y1008) and STAT3 (Tyr705) in WRO 82‐1 cells compared with untreated cells (all *P* < 0.05). Nevirapine with 200 μM reduced phosphorylation expression of JAK2 (Y1007+Y1008) and STAT3 (Tyr705) by 56.0% and 46.1%, respectively (all *P* < 0.05).

## Discussion

Nevirapine plays a vital role in differentiation of progenitor cells or redifferentiation of transformed cells, and in inhibiting genesis and progression of human tumors,^13^, ^14^, ^15^, ^16^, ^17^ but there has not previously been any published research on thyroid cancer. We hypothesized that nevirapine could inhibit the invasion and metastasis of thyroid cancer and, as expected, our results showed that nevirapine suppressed the migration and invasion in dedifferentiated thyroid cancer cell in a dose‐ and time‐dependent manner. Furthermore, nevirapine could significantly ameliorate tumor metastasis in vivo.

Nevirapine, as an immunomodulator, has been shown to inhibit the secretion of cytokine IL‐6 in HIV‐infected patients.^29^ Accumulating studies suggest that thyroid cancer is also an autoimmune disease.^30^, ^31^, ^32^ We found that nevirapine could inhibit IL‐6 mRNA expression in thyroid cancer by RT‐PCR. To elucidate the mechanism underlying the effects of IL‐6 in thyroid cancer, we assessed the expression of its downstream genes. STAT3, a transcription factor, binds to IL‐6 responsive elements located in the promoter region of various acute‐phase genes^33^ and is indicated to be phosphorylated on its Tyr705 in response to IL‐6.^34^ JAK2, an upstream activator of STAT3 and STAT5, has been identified as an oncogenic protein^35^, ^36^ and upregulated expression is associated with more malignant behavior of tumor cells in a large number of human cancers, such as cancers of the breast, ovary, and prostate.^37^, ^38^, ^39^, ^40^ To determine whether nevirapine could inhibit IL‐6 ‐induced expressions of pJAK2 and pSTAT3 in thyroid cancer, we examined expressions of pJAK2 (Y1007+Y1008) and pSTAT3 (Tyr705) by western blot analysis. Our data showed that nevirapine inhibited distant metastasis of tumor xenografts and phosphorylation of pJAK2 and pSTAT3 proteins in WRO 82‐1 cells, indicating that nevirapine exerts its anticancer effects possibly by inhibiting IL‐6/JAK2/STAT3 signaling pathway.

MMPs play a critical role in tumor invasion and metastasis by degrading the molecules constituting the extracellular matrix (ECM).^19^, ^41^, ^42^ Of the more than 20 known human MMPs, MMP2 and MMP9 seem to play crucial roles in tumor invasion and metastasis due to their ability to degrade the ECM, observed in a variety of cancer cell lines.^43^, ^44^ Here, we detected the effects of nevirapine on MMP‐2 and MMP‐9 expression in WRO 82‐1 cells and tumor xenografts. Western blot analysis showed that MMP2 and MMP9 expression was significantly attenuated in cells in a dose‐ and time‐dependent manner, immunohistochemistry also demonstrated that nevirapine decreased the expression of MMP‐2 and MMP‐9. These results indicated that nevirapine may exert its anti‐invasion and antimetastasis effects by regulating MMP2 and MMP9 expression.

Finally, we successfully constructed tumor xenografts in nude mice, and observed liver metastasis of thyroid carcinoma. Treatment with nevirapine resulted in smaller numbers and volumes in liver metastatic tumor. However, since liver metastases were unintentionally found during the experiment, they are less intuitive than those in nude mice inoculated subcutaneously with WRO 82‐1 cells labeled with luciferase. Improvements will be conducted in further research to explore these mechanisms in depth. Nevertheless, our findings suggest that nevirapine plays a crucial role in attenuating metastasis in thyroid cancer.

There is a limitation for this study. The detailed mechanism of inhibiting migration and invasion by nevirapine in dedifferentiated thyroid cancer cells was not performed in our study, but the next part of our work will be to explore in depth how nevirapine works to inhibit migration and invasion by IL‐3/STAT3 pathway.

In summary, these data suggest that nevirapine has inhibitory effects in human dedifferentiated thyroid cancer by targeting the IL‐6/STAT3 signaling pathway, and our findings demonstrate that nevirapine may be useful as a therapy against human thyroid cancer.

## Disclosure

The authors declare that they have no conflict of interest.
